# Variations in the Thermomechanical and Structural Properties during the Cooling of Shape-Memory R-PETG

**DOI:** 10.3390/polym16141965

**Published:** 2024-07-09

**Authors:** Ștefan-Dumitru Sava, Bogdan Pricop, Radu-Ioachim Comăneci, Nicanor Cimpoeșu, Mihai Popa, Nicoleta-Monica Lohan, Leandru-Gheorghe Bujoreanu

**Affiliations:** Faculty of Materials Science, “Gheorghe Asachi” Technical University of Iași, Blvd. Dimitrie Mangeron 71A, 700050 Iasi, Romania; stefan-dumitru.sava@student.tuiasi.ro (Ș.-D.S.); bogdan.pricop@academic.tuiasi.ro (B.P.); radu-ioachim.comaneci@academic.tuiasi.ro (R.-I.C.); nicanor.cimpoesu@academic.tuiasi.ro (N.C.); mihai.popa@academic.tuiasi.ro (M.P.); nicoleta-monica.lohan@academic.tuiasi.ro (N.-M.L.)

**Keywords:** recycled PETG, 3D printing, shape-memory effect, storage modulus, tensile testing, thermomechanical cycling, cooling effect, fractographs, brittleness

## Abstract

One of the useful features of 3D-printed specimens of recycled polyethylene terephthalate glycol (R-PETG) is the ability to repetitively develop free recovery as well as the work-generating, shape-memory effect. This behavior is enabled by the R-PETG’s capacity to stiffen during cooling, thus allowing for a new temporary shape to be induced. Aiming to devise an explanation for the polymer’s stiffening, in this study, the variation in some of the R-PETG’s parameters during cooling are emphasized and discussed. The evolution of an R-PETG filament’s shape was monitored during room-temperature-bending heating–cooling cycles. Straight-shape recovery and the complete loss of stiffness were observed at the start and the end of heating, respectively, followed by the forced straightening of the filament, performed by the operator, around 40 °C, during cooling. The tests performed by dynamic mechanical analysis disclosed the rise of the storage modulus (E’) after 100 °C heating followed by either liquid-nitrogen- or air-cooling to room temperature, in such a way that E’ was always larger after cooling than initially. Static tests emphasized a peculiar stress variation during a heating–cooling cycle applied in air, within the heating chamber of the tensile testing machine. Tensile-failure tests were performed at −10 °C at a rate of 100 mm/min, with specimens printed at various deposition directions between 10 and 40° to the transversal direction. The specimens printed at 40°, which had the largest ultimate strains, were broken with tensile rates between 100 and 500 mm/min. Deformation rate increase favored the shift from crazing to delamination failure modes. The correlation between the structural changes, the sharp E’ increase on heating, and the stiffening induced by cooling represents a novel approach that enables the use of 3D-printed R-PETG for the fabrication of the active parts of low-priced lightweight resettable actuators.

## 1. Introduction

Glass transition corresponds to the reversible transformation of a hard low-temperature amorphous phase into a soft high-temperature (semi)crystalline one [1]. Consequently, the elasticity modulus markedly decreases during heating and recovers its high values during cooling [2]. On the other hand, glass transition has been identified as the governing mechanism of the shape-memory effect (SME) occurring in polymers [3] which consists of the heating-induced recovery of a permanent shape after cooling and deforming to a temporary one.

The polymer is heated, deformed to a temporary shape, and rapidly cooled, which enables internal stresses to be frozen within the matrix that becomes amorphous. During heating, glass transition occurs, the polymer softens, and the stored stresses cause the recovery of the permanent shape [4].

Aiming to improve the durability and strength of polyethylene terephthalate (PET), polyethylene terephthalate glycol (PETG) was developed. Compared with PET, PETG has higher impact and high-temperature resistance [5], which enabled its use as a raw material for textiles, beverage bottles, packaging materials, and 3D printing filaments [6].

In the particular case of 3D printing, PETG filaments have better mechanical properties than PET filaments [7,8], due to the higher glass transition interval, lower melting temperature, and viscosity of the former [9].

Consequently, various complex parts have been 3D printed from PETG using different additive manufacturing techniques [10,11,12].

In two previous studies, the present authors have reported the occurrence of SMEs in filaments and 3D-printed parts of recycled polyethylene terephthalate (R-PET) [13] and polyethylene terephthalate glycol (R-PETG) [14], along with a systematic and comprehensive analysis of the two materials. In these studies, filament and 3D-printed specimens were bent at room temperature (RT), maintained this temporary shape, and recovered their straight permanent shape during heating to 100 °C. More specifically, R-PET printed parts experienced free-recovery SMEs for up to three consecutive cycles, during which a delay was noticed between the displacement and temperature variations, which were fitted with Boltzmann-type functions with standard errors below 1%. [13]. In the cases of filaments produced from R-PETG pellets and 3D-printed parts obtained from these filaments, both free-recovery and work-generating SMEs were emphasized. [14].

Nevertheless, R-PET experienced an obvious glass transition degradation which did not allow a repetitive and reproducible SME to be obtained [13]. In contrast, R-PETG enabled both repetitive and work-generating SMEs, experiencing a specific work output of [9.981 g × (9.81 m·s^−2^) × 1 mm]/ 0.1724 g ≈ 0.568 J/kg. It was pointed out that R-PETG filaments could be straightened during cooling after they completely lost their stiffness during heating [14].

These peculiar features of PETG allowed for the development of specific applications with highly controllable self-coiling and tensile shape-memory behavior [15] as an effect of accurate tuning of the programming temperature [16]. Among the experimental approaches used to improve the mechanical properties of PETG printed specimens, one can mention aging-induced relaxation [17], accurate control of overlapping ratio parameters [18], spatial orientation of layers [19], or the development of complex architectures with honeycomb structures [20].

**Figure 1:**
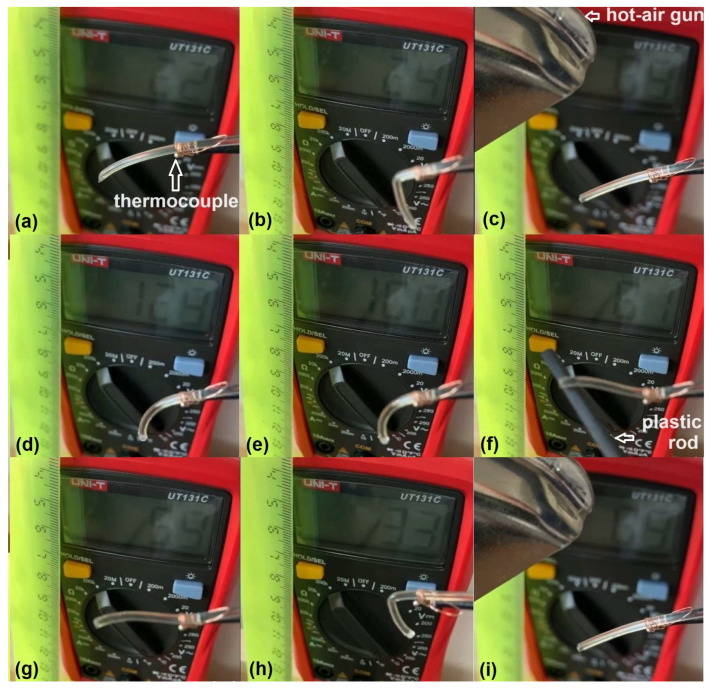
Typical evolution of an R-PETG filament during free-recovery SME thermomechanical cycling: (**a**) permanent shape at 22 °C; (**b**) inducing a temporary bent shape at 24 °C (heating caused by contact with operator’s hand); (**c**) permanent shape recovery during heating at 69 °C; (**d**) stiffness loss at 128 °C; (**e**) stiffness is retained during cooling (here at 100 °C); (**f**) straightening at 61 °C, during cooling, while the specimen still has no stiffness; (**g**) stiffness regained at 59 °C, during cooling; (**h**) a new temporary bent shape is induced at 33 °C; and (**i**) permanent shape recovery during heating at 69 °C.

This behavior of an R-PETG filament during repetitive free-recovery SME cycling is illustrated in Figure 1 by nine frames taken by cinematographic analysis [21].

The R-PETG filament, to which a thermocouple was fastened (Figure 1a), was manually bent at room temperature (Figure 1b,h), heated with a hot-air gun (Figure 1c,d,i), and straightened with a plastic rod during cooling (Figure 1f). During heating, after first recovering its permanent straight shape, as shown in Figure 1c, the filament completely lost its stiffness at 128 °C (Figure 1d). During cooling, the filament still had no stiffness at 100 °C (Figure 1e) and had to be straightened with a plastic rod at 61 °C (Figure 1f). Figure 1g shows that the filament regained its stiffness when the temperature dropped further to 59 °C and the plastic rod could be withdrawn. A new bent shape could be induced at room temperature (Figure 1h) and the procedure could be resumed, since the filament recovered its shape during the second heating (Figure 1i).

It is this intriguing stiffness recovery that has enabled the repetitive thermomechanical cycling of R-PETG by free-recovery SMEs. Conversely, the stiffness of the amorphous shape at room temperature must not increase too much or it will impede the development of a temporary shape different from the permanent one [22].

Recycling the huge amount of PET and PETG waste found in nature has been one of the most challenging demands of the present day. Turning a part of R-PETG into the executive parts of low-priced lightweight actuators has the potential to generate high value for the recycling action. A recent review of the viability of 3D printing in polymer recycling pointed out that this additive manufacturing technique has the potential to replace incineration as the most effective plastic-waste-management technique [23].

Considering that, to the best of our knowledge, there are no reports on PETG’s behavior during cooling, the present study aimed to investigate the evolution of some of the polymer’s structural and thermomechanical parameters during free or controlled temperature decreases. These investigations have the potential to correlate the structural changes induced by cooling with the stiffening observed in 3D-printed R-PETG specimens, enabling their use as active parts of low-priced lightweight resettable actuators. Studying R-PETG’s behavior during cooling represents a novel approach that may contribute to advancement of knowledge of the features of shape-memory polymers.

## 2. Materials and Methods

### 2.1. Materials

Filaments were produced from R-PETG grains manufactured by Selenis Company (Portalegre, Portugal), using an experimental production line previously described in detail [13].

Using the previously detailed values of printing parameters and printer specifications, specimens were printed for both tensile testing and thermal analysis. The former had a “dog-bone” shape, according to [24] type 1BB, with the configuration shown in Figure S1 in the Supplementary Materials. The “dog-bone” specimens were 2 mm-thick, with a total length of 150 mm, lateral fastening shoulders with a length of 20 mm, and a central gauge, 10 mm-wide and 50 mm-long. The latter had a rectangular profile with the dimensions 1 mm × 4 mm × 50 mm. Both types of specimens were printed at a speed of 20–80 mm/s, and were able to provide corresponding characteristics for surface profile and material porosity [25]. Five different angles between the filament’s deposition direction and specimen’s transversal axis were used: 0, 10, 20, 30, and 40° [6].

### 2.2. Testing Methods

The rectangular specimens were subjected to dynamic mechanical analysis (DMA) performed on a NETZSCH DMA 242 Artemis device (Netzsch, Selb, Germany) with a dual cantilever specimen holder. The technical data of the analyzer were explicitly stipulated in our last study [14].

While being bent by 100 μm at a frequency of 1 Hz under an Ar protective atmosphere, the specimens were heated at 3 °C/min up to 100 °C and then cooled to 20 °C, either at 0.5 °C/ min, with free-air flow support (air cooling), or at 3 °C/min, with liquid-nitrogen support (LN cooling).

At this point, it is worth mentioning that uncontrolled cooling plays a special part in the exploitation of the materials’ shape memory since it reproduces more accurately their actual free-air functioning [26].

To analyze the effects of cooling on both the behavior of R-PETG specimens during tensile-failure tests, and the resulting fractographic structure, the specimens were broken with an INSTRON 3382 tensile testing machine with a thermal chamber (Norwood, MA, USA) with cross-head speeds of 100, 200, 300, 400, and 500 mm/min. Deformation speeds larger than 100 mm/min were chosen to reduce the variation in the specimen’s temperature during the test. Using 100 mm/min intervals for the cross-head speed was more effective in emphasizing the deformation rate’s effects on R-PETG behavior during tensile failure at low temperatures and on the resulting fractographic structure.

The machine’s main features have been previously mentioned [14]. To decrease the temperature of the “dog-bone” specimens, the lateral surface of their gauges (with dimensions 2 mm × 10 mm) was directly cooled with DISPO ICE spray (Dispotech, Gordona, Italy) down to −10 °C. The specimens’ temperatures were monitored with a thermocouple and the tests started only after reaching −10 °C. At these large deformation rates, failure occurred in less than 3 s.

The fractured surfaces of “dog-bone” specimens were metalized with a 10 nm thick gold layer, using a LUXOR Au-Pt Coater (APTCO, Berlin, Germany). A fractographic study was performed on a VEGA II LSH TESCAN scanning electron microscope (TESCA, Brno-Kohoutovice, Czech Republic), as previously described [14].

## 3. Results and Discussion

### 3.1. DMA Study

**Figure 2:**
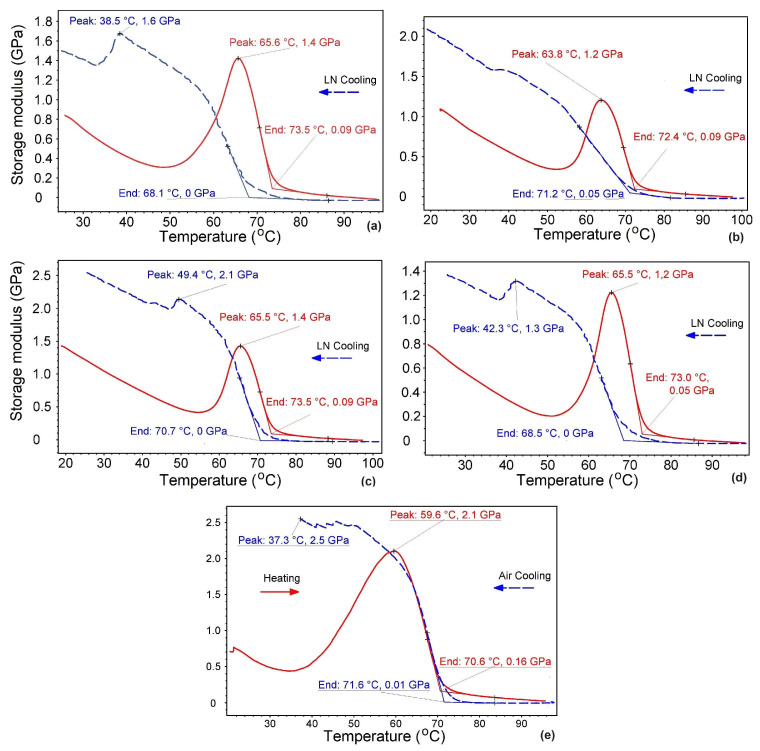
Typical variations in storage modulus during a heating (continuous line)–cooling (dotted line) cycle, of rectangular R-PETG specimens printed at various deposition angles to the transversal direction: (**a**) 10° with LN cooling; (**b**) 20° with LN cooling; (**c**) 30° with LN cooling; (**d**) 40° with LN cooling; and (**e**) 40° with air cooling.

The stiffness recovery during cooling, and the typical variations in storage modulus, E’, during a heating–cooling cycle between 20 and 100 °C, applied to rectangular specimens printed at various angles, are illustrated in Figure 2.

The first DMA experiments were performed with LN-supported cooling, as shown in Figure 2a–d. During heating, a marked stiffening caused by storage-modulus increase was observed. This was almost double the values reported by Pricop et al. for R-PET [13]. This phenomenon could be a logical explanation for the capacity of R-PETG to develop work-generating SMEs [14]. In Figure 2, the storage modulus reached maximum values between 1.2 and 2.1 GPa, after which it sharply decreased. This is a typical phenomenon associated with glass transition [3].

The thermal range of glass transition during heating was determined by the DMA device’s software as 59.6–73.5 °C. Continuing the heating beyond the upper glass transition temperatures caused the storage modulus to decrease to 0.05–0.09 GPa, which explains the loss of stiffness noticed in Figure 1d,e during free-recovery SME thermomechanical cycling. Similar stiffness losses during heating of PETG were confirmed by other experimental methods [27].

During cooling, between 68.1 and 71.2 °C, the storage modulus started to abruptly increase, reaching maximum values between 1.4 and 2.5 GPa, at 20 °C. These storage-modulus values were larger than those determined at the beginning of heating for specimens printed at all of the four deposition angles. In addition, the cooling portions displayed a storage-modulus maximum located between 1.3 and 2.1 GPa which is higher than the maximum values reached during heating. Figure 2e illustrates storage-modulus variation during free-air-flow-supported cooling. In this case, the storage-modulus increase started at a higher temperature, 71.6 °C, compared with liquid-nitrogen-supported cooling. This difference can be ascribed to the glass transition effects of lower cooling rates, induced by free-air-flow-supported cooling, compared with higher free rates caused by liquid-nitrogen-supported cooling [28].

It would be interesting to know how much the storage modulus would increase during a heating–cooling cycle. These experiments are under development and their results will be described in a subsequent report.

Another variation tendency refers to the evolution of the storage modulus with the increase in the angle between the specimen’s transversal direction and layer deposition direction. Even if this tendency was less obvious in the present study, it has been argued that the lowest deposition angles cause the highest storage-modulus values [14]. This tendency was explained as follows: With the increase in the angle to the transversal direction, an augmentation occurs of the length of the fibers with fixed ends, by the specimen’s lateral edges, that were bent by the pushrod. Increasing the length of a solid with fixed ends subjected to bending under constant force would normally cause an increase in bending deflection due to the proportionality between deflection and fibre length [14]. These arguments are based on the commun formula of maximum bending deflection:

(1) $$\delta_{max}=\frac{FL^{3}}{48\;E^{\prime }I}\;,$$

where: *F* is the bending force, *L* is the bending length, *E′* is the storage modulus, and *I* is the moment of inertia.

In conclusion, increasing the angle to the transversal direction caused an increase in bending deflection $\delta_{max},$ which finally led to a decrease in the storage modulus.

### 3.2. Tensile Tests

In the previous section, we described how the R-PETG specimens became stiffer after a heating–cooling cycle of dynamic bending. To further analyze the behavior of these specimens during cooling, while being statically loaded, tensile tests were performed both during heating–cooling and in the cooled condition.

**Figure 3:**
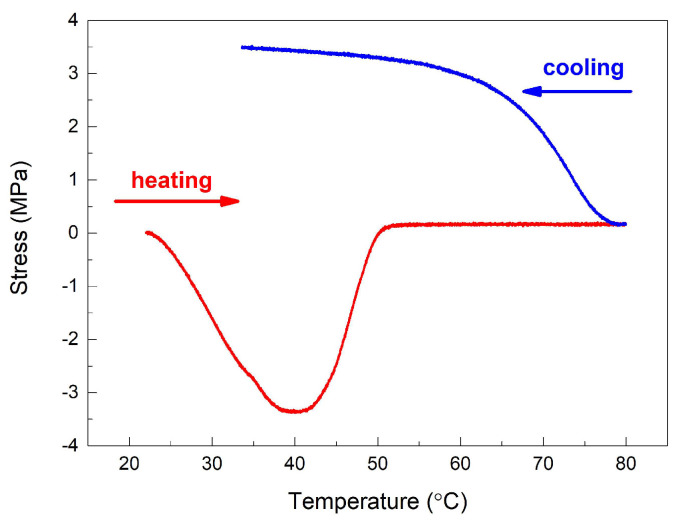
Stress–strain variation of a “dog-bone” specimen, printed at 0°, during a heating–cooling cycle performed in the heating chamber of the tensile testing machine.

Figure 3 illustrates the behavior of a “dog-bone” specimen, printed at 0°, which was fastened in the grips of the tensile testing machine, without being loaded, and subjected to a heating–cooling cycle up to 80 °C.

At the start of the heating, while the temperature increased up to 40 °C, the specimen experienced thermal expansion, thus subjecting the grips to compression stress. As it was heated beyond this temperature, the specimen started to gradually lose its stiffness and stress decreased to zero at 50 °C. Further temperature increase did not cause any stress variation because the specimen had completely lost its stiffness. After reaching 80 °C, the heating was interrupted and the fan of the heating chamber was turned on. It is interesting to note that stress immediately started to increase, reaching 3.5 MPa at about 34 °C, where it stabilized. So, without being mechanically loaded, the “dog-bone” R-PETG specimen, printed along its transversal direction, developed a 3.5 MPa stress after being heated from 20 °C to 80 °C and cooled back to 20 °C.

This evolution confirms, once more, that 3D-printed R-PETG specimens have the capacity to regain their stiffness during cooling, after completely losing it at the end of the previous heating, whether subjected to static or dynamic loading.

**Figure 4:**
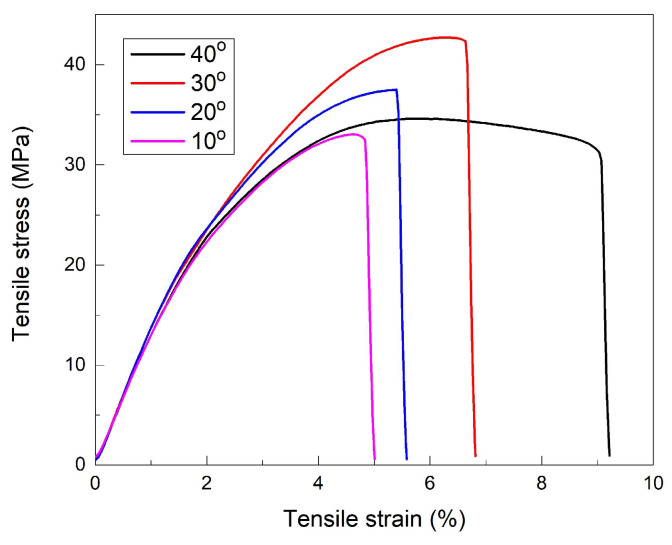
Tensile-failure curves of “dog-bone” specimens printed at different angles to the transversal direction, loaded at room temperature, at a deformation rate of 100 mm/min.

To reveal the tensile behavior changes caused by cooling, the specimens’ gauges were refrigerated at −10 °C and broken at a rate of 100 mm/min, as shown in Figure 4.

As previously pointed out, for deposition angles between 10 and 30° to the transversal direction, both ultimate stress and strain increased due to the simultaneous augmentation of both the number of adjacent layers that were simultaneously deformed and their bonding area. However, at 40° ultimate stress decreased and a marked delamination phenomenon was noticed, suggesting that cohesion was lost between successive layers when their bonding area increased too much [14].

The deformation rate was 100 times larger than in our previous study on R-PETG (where the cross-head speed was 1 mm/min and tensile testing was performed at room temperature). In addition, the testing temperature was about 30 degrees lower, in the present case [14]. For this reason, the R-PETG specimens experienced a higher brittleness degree, failure rate, and larger ultimate stresses but lower strains [29]. Nevertheless, both at room temperature and at −10 °C, the largest ultimate strains were obtained with the specimens printed at 40°, owing to the simultaneous increase in the number of deformed layers and their bonding area [30,31].

**Figure 5:**
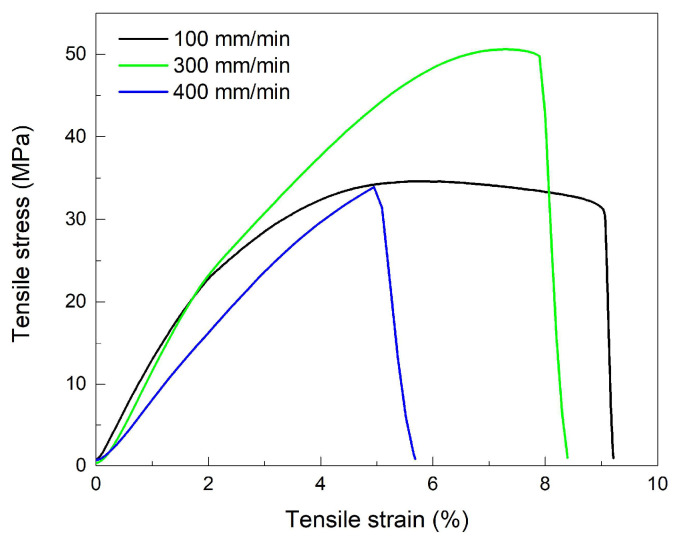
Tensile-failure curves of “dog-bone” specimens printed at 40° to the transversal direction, loaded at room temperature, at three deformation rates.

For a more detailed insight into the effects of cooling on the structural changes, several “dog-bone” R-PETG specimens, printed at 40° to the transversal direction, were subjected to tensile-failure tests, while being refrigerated to −10 °C and deformed with increasing cross-head speeds. The resulting tensile-failure curves are displayed in Figure 5.

As expected, with the increase in the deformation rate, the ultimate strain decreased and this evolution could be ascribed to the progressive hindering of the molecular movements of polymeric chains [32]. The specimens that failed at 100 mm/min and 300 mm/min experienced a certain amount of necking, emphasized by a small stress decrease before failure [33]. On the other hand, the specimen that failed at 400 mm/min did not experience any stress decrease, which is evidence of a brittle behavior [34]. The tensile-failure parameters, determined from Figure 4 and Figure 5, are summarized in Table 1.

According to [24], the Young’s modulus of 1BB type specimens is determined by the “segment method” as E = (s_2_ − s_1_)/(e_2_ − e_1_) where s is stress and e is strain (e_1_ = 0.0005 and e_2_ = 0.0025).

It was expected that all these changes in tensile mechanical properties, caused by both cooling temperature and deformation rate augmentation, would be accompanied by structural changes. In order to investigate this possible connection, the broken surfaces of the gauges of tested specimens were metalized by deposition of a thin Au layer, and analyzed by SEM observations.

### 3.3. SEM Analysis

Compared with our previous report that analyzed the cross-sections of 3D-printed R-PETG specimens that failed at room temperature in tension with a cross-head speed of 1 mm/min [14], the SEM fractographs of the present study were characterized by a more prominent surface relief which can be associated with a more brittle behavior [35].

To better emphasize the effects of large cross-head speeds and cryogenic cooling temperatures on the structure of R-PETG specimens, the specimens were transported to the metallization equipment in an insulated refrigerated container. After metallization, SEM observations were performed at magnifications ranging between 50:1 and 2500:1.

**Figure 6:**
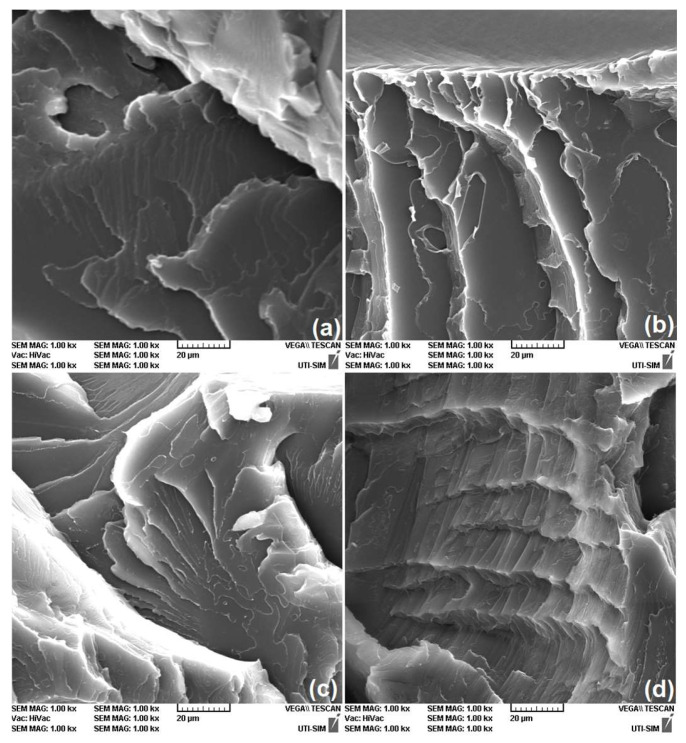
Typical SEM fractographs, printed at various deposition angles to the transversal direction, of the cross-sections of R-PETG “dog-bone” specimens that, according to Figure 4, failed at −10 °C: (**a**) 10°; (**b**) 20°; (**c**) 30°; and (**d**) 40°.

Typical examples are shown in Figure 6, at a magnification of 1000:1 and printed at various deposition angles to the transversal direction, of the cross-sections of the specimens that, according to Figure 4, had failed.

Figure 4 shows that, with the increase in the deposition angle, both the ultimate strain and the ductile behavior (emphasized by the presence of necking) became more prominent. These aspects are reflected in the fractographs in Figure 6. With the increase in the deposition angle, more and more consecutive failure layers are noticeable which can explain the increase in plasticity [36]. Compared with our previous SEM micrographs, recorded on R-PETG specimens that were broken in tension at room temperature and 1 mm/min deformation rate, the images in Figure 6 reveal crack-like, sharply localized bands of plastically deformed material called “crazes” [37].

The characteristic aspect of the crazes is the presence of elongated nanovoids that easily break down to form cracks [38].

**Figure 7:**
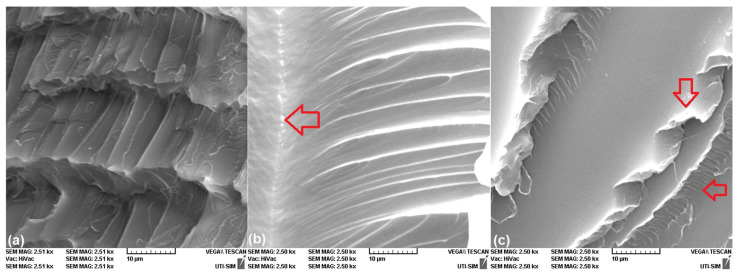
Typical SEM fractographs of the cross-sections of R-PETG “dog-bone” specimens, printed at 40°, that, according to Figure 5, failed at different deformation rates: (**a**) 100 mm/min; (**b**) 300 mm/min; and (**c**) 400 mm/min.

Among the four micrographs from Figure 6, Figure 6d, printed at 40°, displays the largest density of crazes, and further SEM investigations aimed to reveal the influence of cross-head speed during rupture, using the specimens that, according to Figure 5, were broken. The results are summarized in Figure 7.

It is obvious that the crazes were present at a deformation rate of 100 mm/min, in Figure 7a. The fractograph reveals the periodicity of voids that ultimately transformed into cracks. Considering that the crazing phenomenon is rate-dependent [39], tripling the deformation rate changed the aspect of crazes after some of them transformed into cracks. One central crack (marked by a horizontal arrow) is visible in Figure 7b in the specimen broken at 300 mm/min. Further deformation rate increase, to 400 mm/min, caused the intervention of multiple deformation modes, namely modes I (opening) and II (in-plane shearing) [40]. As a consequence, the aspect of the failure surface of the specimen printed at 40° and broken at 400 mm/min changed from a craze-dominated into a delamination-dominated structure [41]. Figure 7c displays ribbons and scarps designated by vertical and horizontal arrows, respectively [42]. The image emphasizes the marked surface relief of this specimen which is in agreement with the brittle character of failure, sustained by the aspect of the curve from Figure 5. These structural metamorphoses suggest that fracture mostly developed at the boundary of adjacent material layers, in an “inter-layer failure mode” [43], being accompanied by an extended degree of bond misalignment [44].

Due to the brittle nature of the final failure of R-PETG specimens that were 3D printed at 40° to the transversal axis and broken at 100 mm/min, the initial deformation mechanism which preceded rupture was crazing [45]. With increasing the deformation rate to 300 and 400 mm/min, the fracturing process proceeded by delamination between adjacent layers of welded material [46].

## 4. Conclusions

The cooling behavior of R-PETG 3D-printed specimens was analyzed both dynamically and statically. The former was performed by DMA tests and the latter by tension. The following conclusions can be drawn:

- The specimens (almost) completely lost their dynamic and static stiffness when heated beyond 70.6–73.5 °C (storage modulus decreased to 0.05–0.16 GPa) and 50 °C (tensile stress decreased to 0 MPa), respectively, and recovered it during subsequent cooling below 71.6–68.1 °C and 80 °C (at the very beginning of cooling), respectively;
- Stiffness recovery enabled the specimens to be deformed into a new temporary shape that supported the continuation of free-recovery SME cycling;
- At the end of a heating–cooling cycle, the stiffness of both dynamically and statically deformed specimens was greater than it had been initially;
- The specimens deformed by tension at −10 °C, with cross-head speeds of at least 100 mm/min, failed at larger ultimate stresses and lower ultimate strains compared with those deformed at room temperature, with a cross-head speed of 1 mm/min, in accordance with previous results reported on the temperature influence on tensile behavior of 3D-printed PETG [45];
- The largest ultimate strains, at a deformation rate of 100 mm/min, were obtained in the specimens printed at the largest angle, 40°, to the transversal deposition direction, which could be an effect of the intensification of the craze phenomenon;
- With increasing deformation rate, up to 400 mm/min, failure occurred by delamination rather than by crazing.

Additional study is necessary to determine the limit to which the storage modulus can increase at the end of the cooling stage of multiple heating–cooling cycles. In this way, the optimal number of functioning cycles can be determined, while considering the necessity to reset the newly deformed temporary shape before triggering a new SME event. These experiments are under development and their results will be subsequently reported. Based on the present results, low-price lightweight resettable actuators can be developed, and be able to deliver work within a given thermal range. Determining the actual design of these actuators is beyond the scope of the present report. In principle, considering R-PETG’s capacity to develop a specific work output of approximately 0.5 J/kg, one idea would be a 3D-printed valve with an even number of circularly disposed radial lamellas which would be forced to open at RT and would close, blocking the fluid’s flow, during heating up to 63 °C [14].

## Figures and Tables

**Table 1:** Mechanical parameters of the tensile-failure curves from Figure 4 and Figure 5.

Angle, °	Rate, mm/min	E, GPa	R_m_, MPa	ε_m_, %
10	100	1.226	42.53	5
20	100	1.339	36.82	5.6
30	100	1.337	42.53	6.8
40	100	1.227	34.6	9.2
40	300	0.755	50.61	8.3
40	400	0.569	33.9	5.8

## Data Availability

Data are contained within the article and Supplementary Materials.

## Supplementary Materials

The following supporting information can be downloaded at: https://www.mdpi.com/article/10.3390/polym16141965/s1, Figure S1: 3D-printed specimen geometry and deposition angles.
