# Supplementary material for: Variations in the Thermomechanical and Structural Properties during the Cooling of Shape-Memory R-PETG
Source: Polymers (Basel). 2024 Jul 9;16(14):1965. doi: 10.3390/polym16141965 (PMC11280682; doi:10.3390/polym16141965)
Supplement: Supplementary file 1 [file polymers-16-01965-s001.zip › polymers-3053739-supplementary.pdf]

Figure S1.

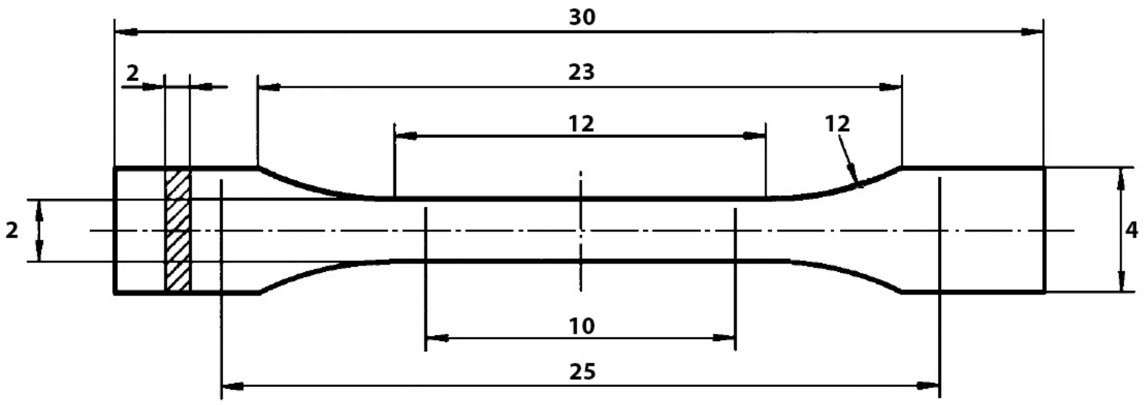

Specimen geometry according to EN ISO 527-2

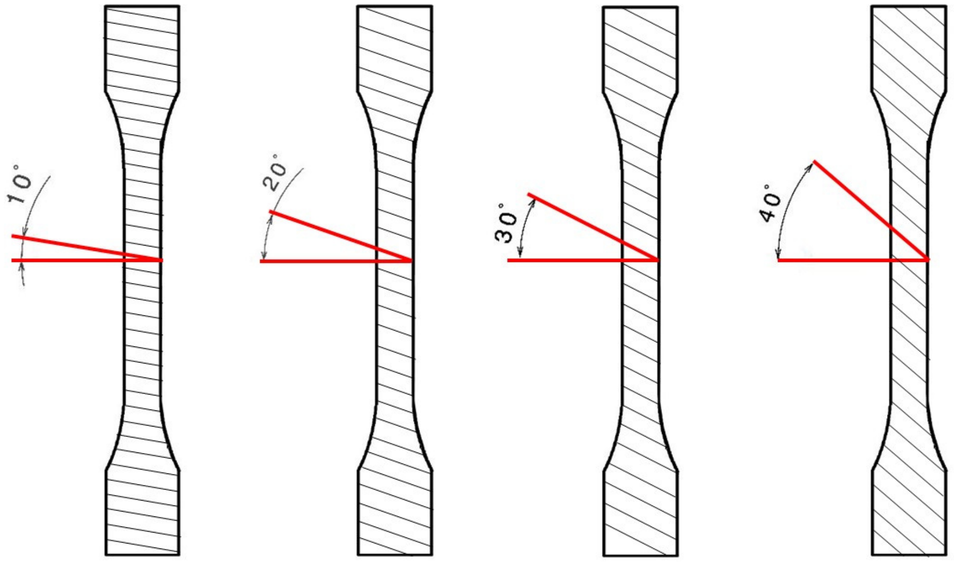

Illustration of the printing angle between transversal direction and filament deposition direction
